# *ADAM12* is an independent predictor of poor prognosis in liver cancer

**DOI:** 10.1038/s41598-022-10608-y

**Published:** 2022-04-22

**Authors:** Shuangqiu Du, Linlin Sun, Yun Wang, Wenhao Zhu, Jialin Gao, Wenjun Pei, Yao Zhang

**Affiliations:** 1 Anhui Province Key Laboratory of Biological Macromolecules Research, Wannan Medical College, Wuhu, 241002 China; 2 Department of Endocrinology, The First Affiliated Hospital of Wannan Medical College, Wuhu, 241002 China

**Keywords:** Cancer prevention, Cancer screening, Tumour biomarkers

## Abstract

Disintegrin and metalloproteinase 12 (ADAM12) is thought to trigger the occurrence and development of numerous tumours, including colorectal, breast, and pancreatic cancers. On the basis of The Cancer Genome Atlas (TCGA) datasets, in this study, the relationship between *ADAM12* gene expression and hepatocellular carcinoma (HCC), the prognostic value of this relationship, and the potential mechanisms influencing HCC development were evaluated. The results showed that the *ADAM12* gene was significantly and highly expressed in liver cancer tissue. The high expression of the *ADAM12* gene in liver cancer tissue significantly and positively correlated with T stage, pathological stage, and residual tumour. Kaplan–Meier and Cox regression analyses revealed that *ADAM12* gene expression is an independent risk factor influencing the prognosis of patients with liver cancer. Pathway analyses of ADAM12 in HCC revealed ADAM12-correlated signalling pathways, and the expression level of ADAM12 was associated with immune cell infiltration. In vitro experiments demonstrated that the expression level of ADAM12 in Huh-7 and Hep3B cells was significantly higher than that in other HCC cells. ShRNA transfection experiments confirmed that the expression levels of TGF-β and Notch pathway-related proteins were significantly decreased. An EdU cell proliferation assay showed that a low level of *ADAM12* gene expression significantly inhibited the proliferative activity of HCC cells. Cell cycle experiments showed that low ADAM12 expression blocked the G1/S phase transition. Overall, this research revealed that high *ADAM12* gene expression implies a poor prognosis for patients with primary liver cancer. In addition, it is a potential indicator for the diagnosis of liver cancer.

## Introduction

Liver cancer is a complex disorder and one of the most prevalent malignant tumours in the digestive system. It is caused by multiple factors, and the primary pathological type is hepatocellular carcinoma (HCC)^1,2^. According to 2018 global cancer statistics, liver cancer is the 4th leading cause of cancer death^3^. Liver cancer has no significant clinical symptoms during the early stages; it is often diagnosed in the intermediate or late stage, at which point, it is highly malignant. The recurrence rate is high. In the past, conventional or single treatment often increased recurrence risk due to residual liver tissue and poor patient tolerance of chemoradiotherapy^4,5^. In recent years, emerging therapeutic methods, including molecular targeted therapeutic drugs and immunity-inducing drugs, have displayed better therapeutic effects and weaker side effects in advanced hepatocellular carcinoma than traditional therapies^6,7^.

Disintegrin and metalloproteinases (ADAMs) are transmembrane glycoproteins anchored to the cell membrane that belong to the Metzincin superfamily of Zn^2+^-dependent metalloproteinase enzymes^8–10^. To date, as many as 40 members of the ADAM protein family have been identified, and they have been shown to regulate cell phenotypes through their effects on cell adhesion, migration, proteolysis, and signal transduction^10–12^. In recent years, several studies have shown that this family promotes the occurrence and development of tumours by targeting cell development, regulating the inflammatory response, and releasing membrane-bound proteins^13,14^.

The *ADAM12* gene is generated by ADAM splicing; is primarily found in the bone and cartilage; and is expressed in the brain, liver, heart, and muscles^15–17^. The human *ADAM12* gene is located on chromosome 10q26.3, and in tumour cell lines, it is expressed in two different spliced forms^16^. Additionally, several studies have shown that the expression of the *ADAM12* gene is upregulated in pancreatic, colorectal, gastric, lung, and breast cancers and leads to poor prognosis^17–21^. Notably, the *ADAM12* gene is a binding partner of the activated protease C receptor during liver fibrosis, and both ADAM12 and protease C are highly expressed in liver cancer and liver fibrosis^22^. Liver fibrosis and cirrhosis caused by chronic hepatitis B are important risk factors for liver cancer^23,24^; therefore, ADAM12 is closely related to liver injury and liver cancer. Nonetheless, reports on ADAM12 in primary liver cancer remain rare. Although studies have suggested that the *ADAM12* gene may be related to the invasion and progression of liver cancer cells^25^, the potential mechanisms and clinical relevance of these actions remain to be clarified.

In this study, we evaluated the contribution of *ADAM12* gene expression to the prognosis of liver cancer using liver cancer samples obtained from the TCGA database. Moreover, we clarified the biological pathway and mechanism of the *ADAM12* gene implicated in the regulation of the occurrence and development of liver cancer via gene enrichment analysis (GSEA) and immune cell invasion analysis. Our findings will help in the discovery of novel prognostic biomarkers and prediction of the potential molecular mechanisms influencing the prognosis of liver cancer.

## Results

### Clinical features of patients with hepatocellular carcinoma

The clinical data used to characterize 374 patients with hepatocellular carcinoma were downloaded from the TCGA database and divided into high and low groups based on the median *ADAM12* gene expression level. Comprehensive information on these clinical data is provided in Table 1.

**Table 1:** TCGA liver cancer patient characteristics.

Characteristic	Low expression of ADAM12	High expression of ADAM12	p
n	187	187	
**T stage, n (%)**			**0.007**
T1	107 (28.8%)	76 (20.5%)	
T2	42 (11.3%)	53 (14.3%)	
T3	34 (9.2%)	46 (12.4%)	
T4	3 (0.8%)	10 (2.7%)	
**N stage, n (%)**			0.354
N0	133 (51.6%)	121 (46.9%)	
N1	1 (0.4%)	3 (1.2%)	
**M stage, n (%)**			1.000
M0	139 (51.1%)	129 (47.4%)	
M1	2 (0.7%)	2 (0.7%)	
**Pathologic stage, n (%)**			**0.021**
Stage I	103 (29.4%)	70 (20%)	
Stage II	39 (11.1%)	48 (13.7%)	
Stage III	36 (10.3%)	49 (14%)	
Stage IV	2 (0.6%)	3 (0.9%)	
**Gender, n (%)**			**0.047**
Female	51 (13.6%)	70 (18.7%)	
Male	136 (36.4%)	117 (31.3%)	
**Age, n (%)**			0.196
≤ 60	82 (22%)	95 (25.5%)	
> 60	105 (28.2%)	91 (24.4%)	
**Residual tumour, n (%)**			**0.023**
R0	174 (50.4%)	153 (44.3%)	
R1	4 (1.2%)	13 (3.8%)	
R2	1 (0.3%)	0 (0%)	
**Histological grade, n (%)**			0.173
G1	34 (9.2%)	21 (5.7%)	
G2	89 (24.1%)	89 (24.1%)	
G3	55 (14.9%)	69 (18.7%)	
G4	7 (1.9%)	5 (1.4%)	
**Vascular invasion, n (%)**			0.276
No	115 (36.2%)	93 (29.2%)	
Yes	53 (16.7%)	57 (17.9%)	
**OS event, n (%)**			**0.013**
Alive	134 (35.8%)	110 (29.4%)	
Dead	53 (14.2%)	77 (20.6%)	
**DSS event, n (%)**			**0.019**
Alive	154 (42.1%)	133 (36.3%)	
Dead	30 (8.2%)	49 (13.4%)	

### ADAM12 is highly expressed in liver cancer tissues

**Figure 1:**
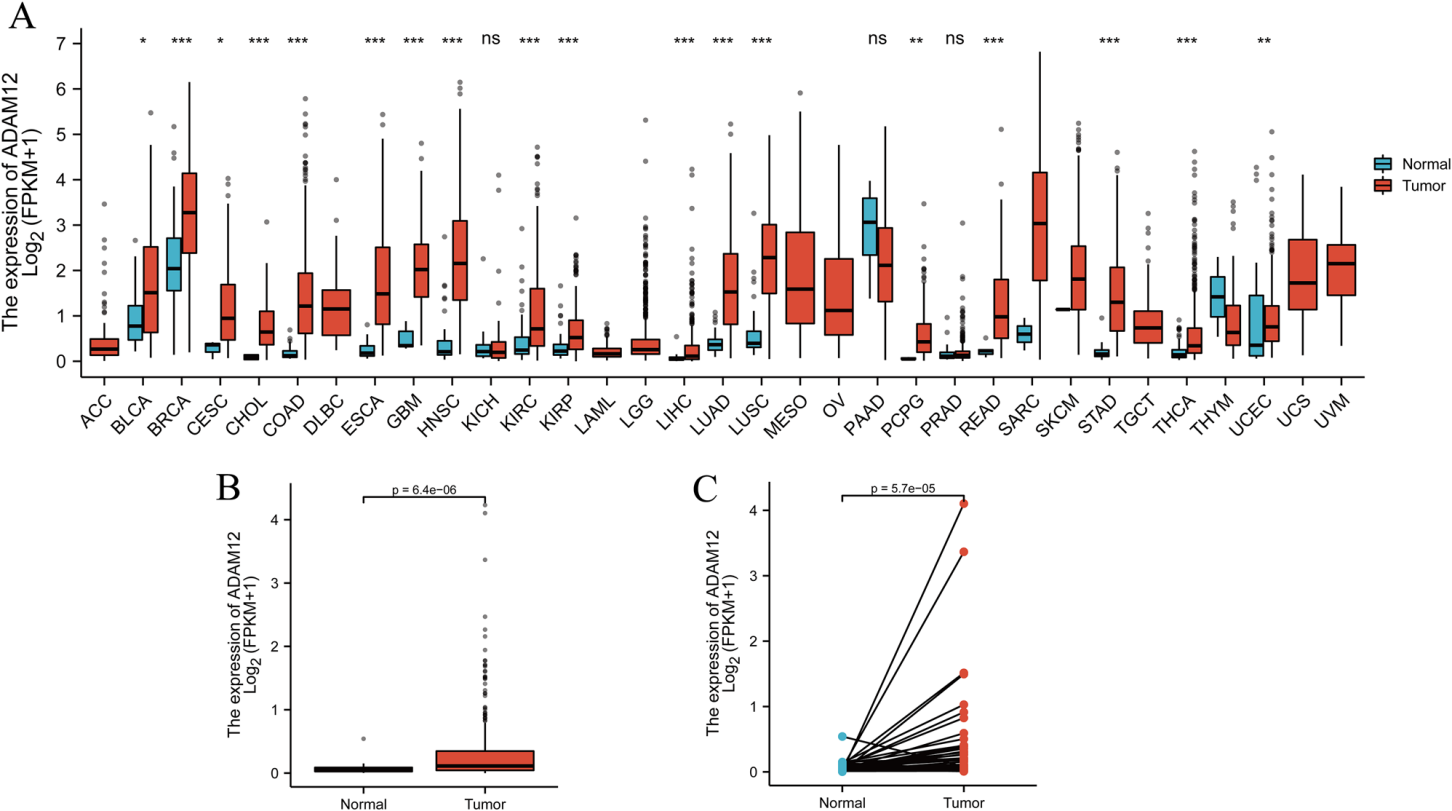
ADAM12 expression was significantly higher in liver cancer tissues than in adjacent normal tissues. (**A**) Increased or decreased ADAM12 expression in different cancers compared with that in normal tissues in the TCGA database. (**B**) ADAM12 expression was significantly higher in cancer tissues than in normal tissues. (**C**) ADAM12 expression was significantly higher in liver cancer tissues than in 50 paired noncancerous adjacent tissues. *p < 0.05, **p < 0.01 and ***p < 0.001 indicate significance.

On the basis of TCGA data, the expression differences in *ADAM12* gene mRNA in different tumour tissues and normal tissues were analysed by Wilcoxon signed-rank test (Fig. 1A). The results revealed that the *ADAM12* gene was expressed in various tumours. In hepatocellular carcinoma (LIHC) and cholangiocarcinoma (CHOL), significant differences were noted between tumour tissue and normal tissue. In hepatocellular carcinoma, the expression of the *ADAM12* gene in tumour tissues was significantly higher than that in normal tissues (*p* = 6.4e−06) (Fig. 1B). Subsequently, Wilcoxon signed-rank test was performed to analyse the paired sample data of ADAM12 expression in 50 cases of liver cancer and adjacent tissues. The results indicated that the expression of ADAM12 in normal tissues was significantly lower than that in cancer tissues (*p* = 5.7e−05) (Fig. 1C).

### Relationship between *ADAM12* gene expression and clinicopathologic features in patients with primary liver cancer

**Figure 2:**
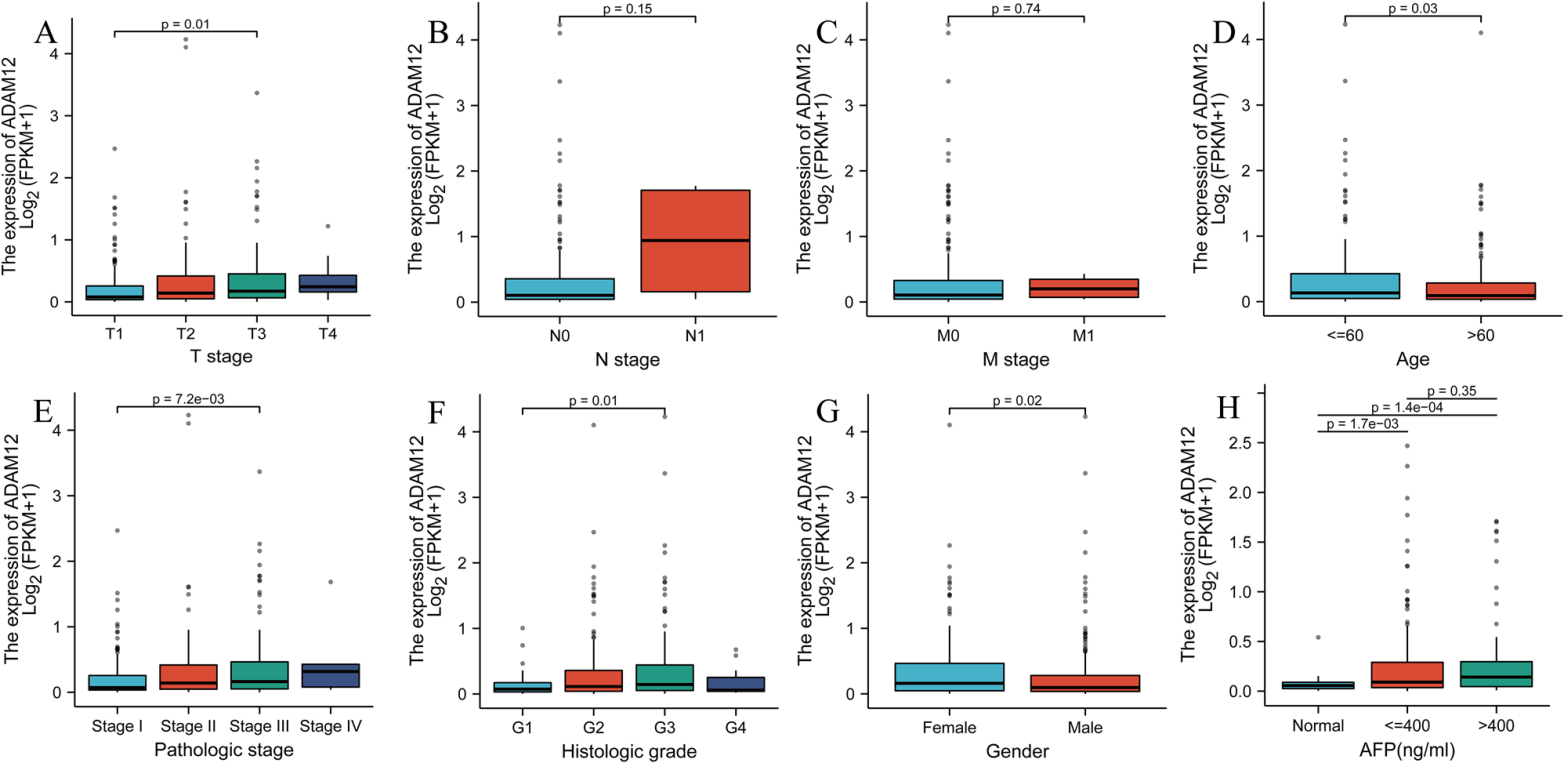
Relationship between ADAM12 expression and clinicopathological characteristics. (**A–C**) TNM classification, (**D**) age, (**E**) pathological stage, (**F**) histological grade, (**G**) sex and (**H**) AFP expression level. TCGA, The Cancer Genome Atlas; AFP, alpha fetoprotein; T, topography distribution; N, lymph node metastasis; M, distant metastasis. *p < 0.05, **p < 0.01 and ***p < 0.001 indicate significance.

The Wilcoxon signed-rank test and logistic regression analysis were performed to evaluate the relationship between *ADAM12* gene expression and clinicopathological variables in patients with hepatocellular carcinoma. The expression of the *ADAM12* gene in hepatocellular carcinoma was significantly correlated with T stage (*p* = *0.01*), age (*p* = *0.03*), sex (*p* = 0.02), pathological stage (*p* = 7.2e−03), and histological grade (*p* = 0.01) (Fig. 2). Thereafter, the relationship between *ADAM12* gene expression and the clinicopathological characteristics of the HCC patients was analysed through univariate logistic regression. The results showed that high expression of the *ADAM12* gene was significantly correlated with T stage, clinicopathological stage, sex, and ethnicity; nevertheless, no significant difference was found between *ADAM12* gene expression and clinicopathological variables, including N stage, M stage, histological grade, vascular invasion (Table 2).

**Table 2:** ADAM12 expression correlated with clinical pathological characteristics (logistic regression).

Characteristics	Total (N)	Odds ratio (OR)	P value
T stage (T2 & T3 & T4 vs. T1)	371	1.943 (1.288–2.943)	**0.002**
N stage (N1 vs. N0)	258	3.298 (0.416–67.150)	0.304
M stage (M1 vs. M0)	272	1.078 (0.128–9.087)	0.941
Pathological stage (Stage III & Stage IV & Stage II vs. Stage I)	350	1.911 (1.252–2.931)	**0.003**
Tumour status (with tumour vs. tumour-free)	355	1.267 (0.832–1.933)	0.270
Age (> 60 vs. ≤ 60 years)	373	0.748 (0.497–1.124)	0.163
Gender (male vs. female)	374	0.627 (0.403–0.969)	**0.036**
Histological grade (G3 & G2 & G4 vs. G1)	369	1.748 (0.979–3.185)	0.062
Vascular invasion (yes vs. no)	318	1.330 (0.837–2.117)	0.228
Race (White & Black or African American vs. Asian)	362	1.537 (1.014–2.338)	**0.043**
Residual tumour (R1 & R2 vs. R0)	345	2.957 (1.088–9.389)	**0.044**

Categorical dependent variable, greater or less than the median expression level. T, topography distribution; N, lymph node metastasis; and M, distant metastasis.

### Clinical significance of *ADAM12* gene expression in the prognosis of liver cancer

**Figure 3:**
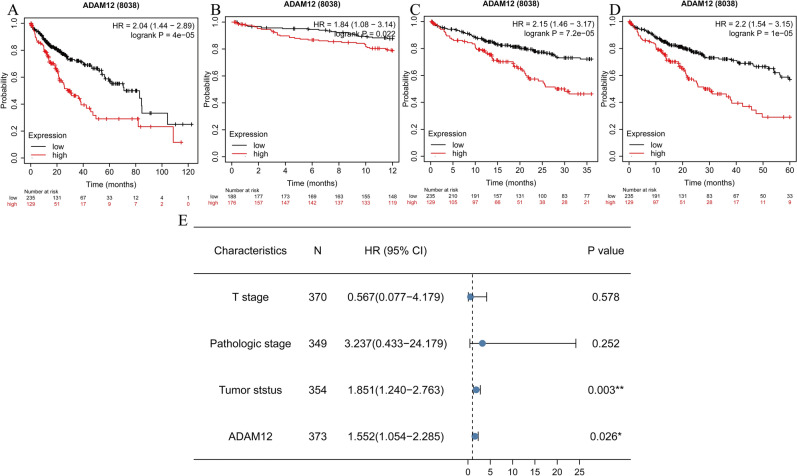
Survival outcomes and multivariate analysis. (**A**) The effect of ADAM12 expression on overall survival in liver cancer patients in the TCGA cohort. The median score was used to categorize patients into high expression and low expression groups. OS analysis of liver cancer patients with high and low ADAM12 expression for (**B**) 12 months (1 year), (**C**) 36 months (3 years), and (**D**) 60 months (5 years). (**E**) Forest map showing the results of the multivariate analysis. *p < 0.05, **p < 0.01 and ***p < 0.001 indicate significance.

Furthermore, the Kaplan–Meier Plotter database was used to analyse the clinical significance of *ADAM12* gene expression in the prognosis of liver cancer. The results showed that the overall survival time of the group with high *ADAM12* gene expression was shorter than that of the group with low *ADAM12* gene expression (*p* = 4e−05) (Fig. 3A). The mean OS time in the low ADAM12 expression group at 12 (1 year), 36 (3 years), and 60 months (5 years) was significantly longer than that in the high expression group (Fig. 3B–D). A univariate Cox regression analysis revealed that *ADAM12* gene expression was a high-risk factor for HCC (HR, 1.818; CI, 1.280–2.582; *p* < 0.001) (Table 3). A multivariate Cox regression analysis showed that high *ADAM12* gene expression was an independent prognostic factor related to OS (HR, 1.552; CI, 1.054–2.285; *p* = 0.026) (Fig. 3E).

**Table 3:** Univariate analysis and multivariate analyses of liver cancer patient overall survival.

Characteristics	Total (N)	Univariate analysis		Multivariate analysis	
		Hazard ratio (95% CI)	P value	Hazard ratio (95% CI)	P value
T stage (T2 &T3 & T4 vs. T1)	370	2.126 (1.481–3.052)	** < 0.001**	0.567 (0.077–4.179)	0.578
N stage (N1 vs. N0)	258	2.029 (0.497–8.281)	0.324		
M stage (M1 vs. M0)	272	4.077 (1.281–12.973)	**0.017**		
Pathological stage (Stage II & Stage III & Stage IV vs. Stage I)	349	2.090 (1.429–3.055)	** < 0.001**	3.237 (0.433–24.179)	0.252
Age (> 60 vs. ≤ 60 years)	373	1.205 (0.850–1.708)	0.295		
Gender (male vs. female)	373	0.793 (0.557–1.130)	0.200		
Tumour status (with tumour vs. tumour-free)	354	2.317 (1.590–3.376)	** < 0.001**	1.851 (1.240–2.763)	**0.003**
Histological grade (G2 & G3 & G4 vs. G1)	368	1.188 (0.721–1.958)	0.499		
ADAM12 (high vs. low)	373	1.818 (1.280–2.582)	** < 0.001**	1.552 (1.054–2.285)	**0.026**

Bold values indicate *p* values < 0.05. T, topography distribution; N, lymph node metastasis; and M, distant metastasis.

### GSEA of the *ADAM12* gene

**Figure 4:**
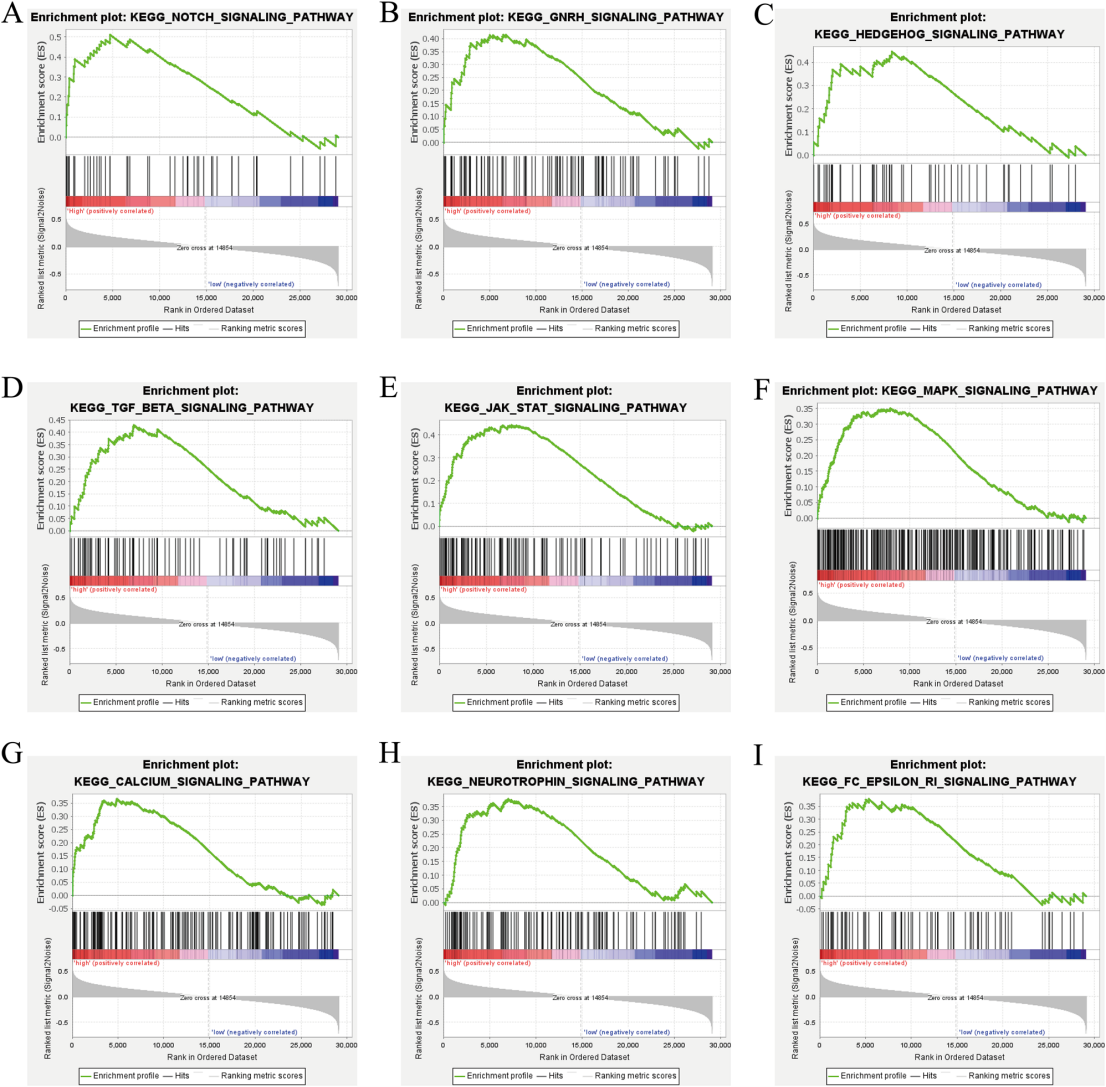
Enrichment plots based on gene set enrichment analysis (GSEA). (**A**) GSEA results showing the Notch signalling pathway. (**B**) GnRH signalling pathway. (**C**) Hedgehog signalling pathway. (**D**) TGF-β signalling pathway. (**E**) JAK/STAT signalling pathway. (**F**) MAPK signalling pathway. (**G**) Calcium signalling pathway. (**H**) Neurotrophin signalling pathway. (**I**) Fc epsilon signalling pathway. NES, normalized enrichment score; ES, enrichment score; FDR, false discovery rate. *p < 0.05, **p < 0.01 and ***p < 0.001 indicate significance.

To explore the possible mechanism of *ADAM12* gene effects in liver cancer, data from the TCGA database were utilized to perform gene set enrichment analysis (GSEA). A Kyoto Encyclopedia of Genes and Genomes (KEGG) pathway enrichment analysis of ADAM12 gene expression samples was performed on the basis of MSigDB enrichment data (c2., Cp., KEGG V7.4, GMT symbols). On the basis of P value, NES value, and FDR value criteria, significantly enriched pathways, including the Notch, GnRH, Hedgehog, TGF-β, JAK/STAT, MAPK, Calcium, Neurotrophin, and Fc epsilon signalling pathways, were found to be enriched in the group with high *ADAM12* gene expression (Fig. 4 and Table 4)^26–28^.

**Table 4:** Gene sets enriched with highly expressed ADAM12.

Gene set name	ES	NES	NOM *P*-val	FDR Q-val
KEGG_NOTCH_SIGNALING_PATHWAY	0.510	1.806	0.007	0.073
KEGG_GNRH_SIGNALING_PATHWAY	0.415	1.694	0.000	0.090
KEGG_HEDGEHOG_SIGNALING_PATHWAY	0.446	1.671	0.022	0.096
KEGG_TGF_BETA_SIGNALING_PATHWAY	0.429	1.667	0.032	0.092
KEGG_JAK_STAT_SIGNALING_PATHWAY	0.443	1.659	0.034	0.092
KEGG_MAPK_SIGNALING_PATHWAY	0.350	1.606	0.008	0.113
KEGG_CALCIUM_SIGNALING_PATHWAY	0.365	1.576	0.023	0.114
KEGG_NEUROTROPHIN_SIGNALING_PATHWAY	0.377	1.544	0.043	0.121
KEGG_FC_EPSILON_RI_SIGNALING_PATHWAY	0.378	1.524	0.040	0.128

Gene sets with an NOM *p* value < 0.05 and an FDR Q-value < 0.25 were considered to be significant. ES, enrichment score; NES, normalized enrichment score; NOM, nominal; and FDR, false discovery rate.

### Relationship between *ADAM12* gene expression and immune cell infiltration

**Figure 5:**
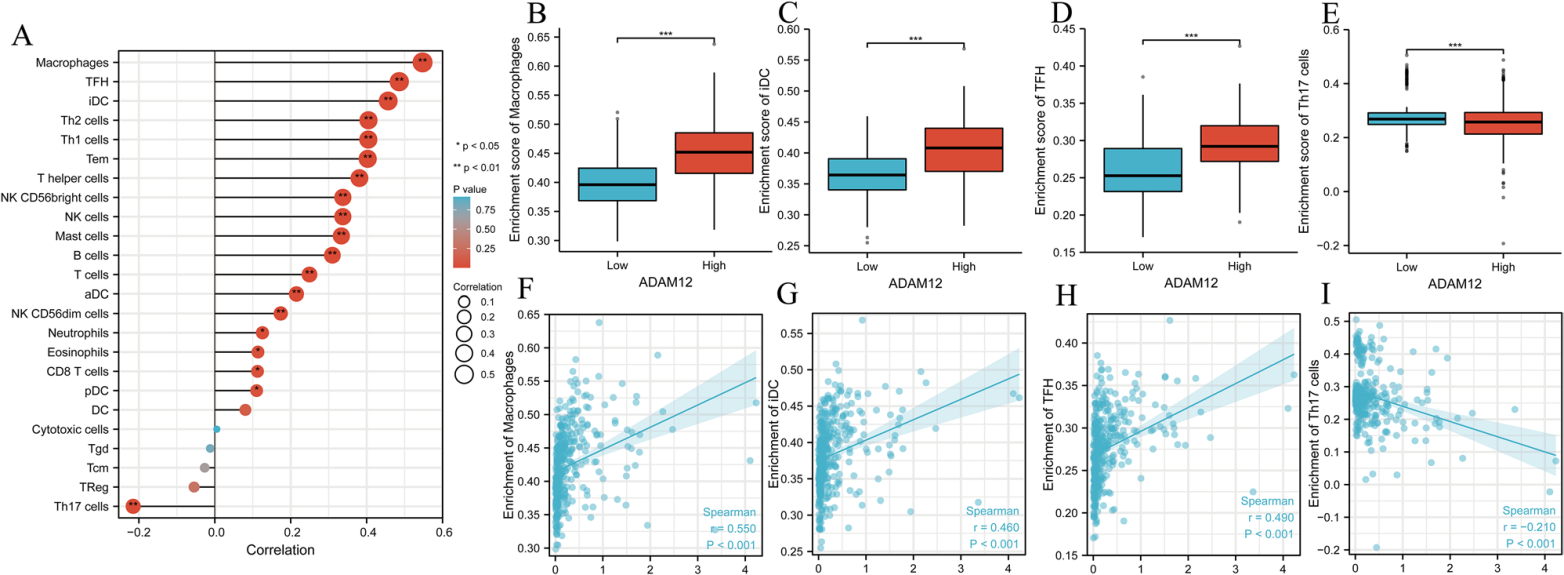
The expression of ADAM12 was related to immune cell infiltration into the tumour microenvironment. (**A**) Association between the ADAM12 expression level and relative abundances of 24 immune cell types. The size of the dots demonstrates the absolute value of Spearman R. (**B–I**) Correlation diagrams and scatter plots indicating the differentiation of macrophages, iDCs, and TFH and Th17 cell infiltration levels between the high and low ADAM12 expression groups. *p < 0.05, **p < 0.01 and ***p < 0.001 indicate significance.

To comprehensively investigate the role of ADAM12 in HCC, we selected the immune infiltrating algorithm (ssGSEA) and Spearman correlation to analyse the association between ADAM12 expression levels and subsets of infiltrating immune cells. The expression of the *ADAM12* gene was found to be positively correlated with macrophages (*p* < 0.01), immature dendritic cells (*p* < 0.01) and follicular T cells (*p* < 0.01), but negatively correlated with helper T cells 17 (*p* < 0.01) (Fig. 5). In addition, ADAM12 was associated with markers of macrophages, immature dendritic cells, follicular T cells and helper T cells (Table 5).

**Table 5:** Correlation analysis between ADAM12 expression and gene markers of immune cells.

Terms	Markers	R	*P* value
Macrophage	IRF5	0.249	< 0.001
	NOS2	0.170	< 0.001
	PTGS2	0.553	< 0.001
	CD163	0.278	< 0.001
	MS4A4A	0.364	< 0.001
	VSIG4	0.391	< 0.001
iDC	CD80	0.529	< 0.001
	CD86	0.503	< 0.001
	ITGAX	0.558	< 0.001
TFH	IL21	0.145	< 0.001
TH17	STAT3	0.415	< 0.001

### Differential expression of ADAM12 in hepatocellular carcinoma tissues and hepatocellular carcinoma cells

**Figure 6:**
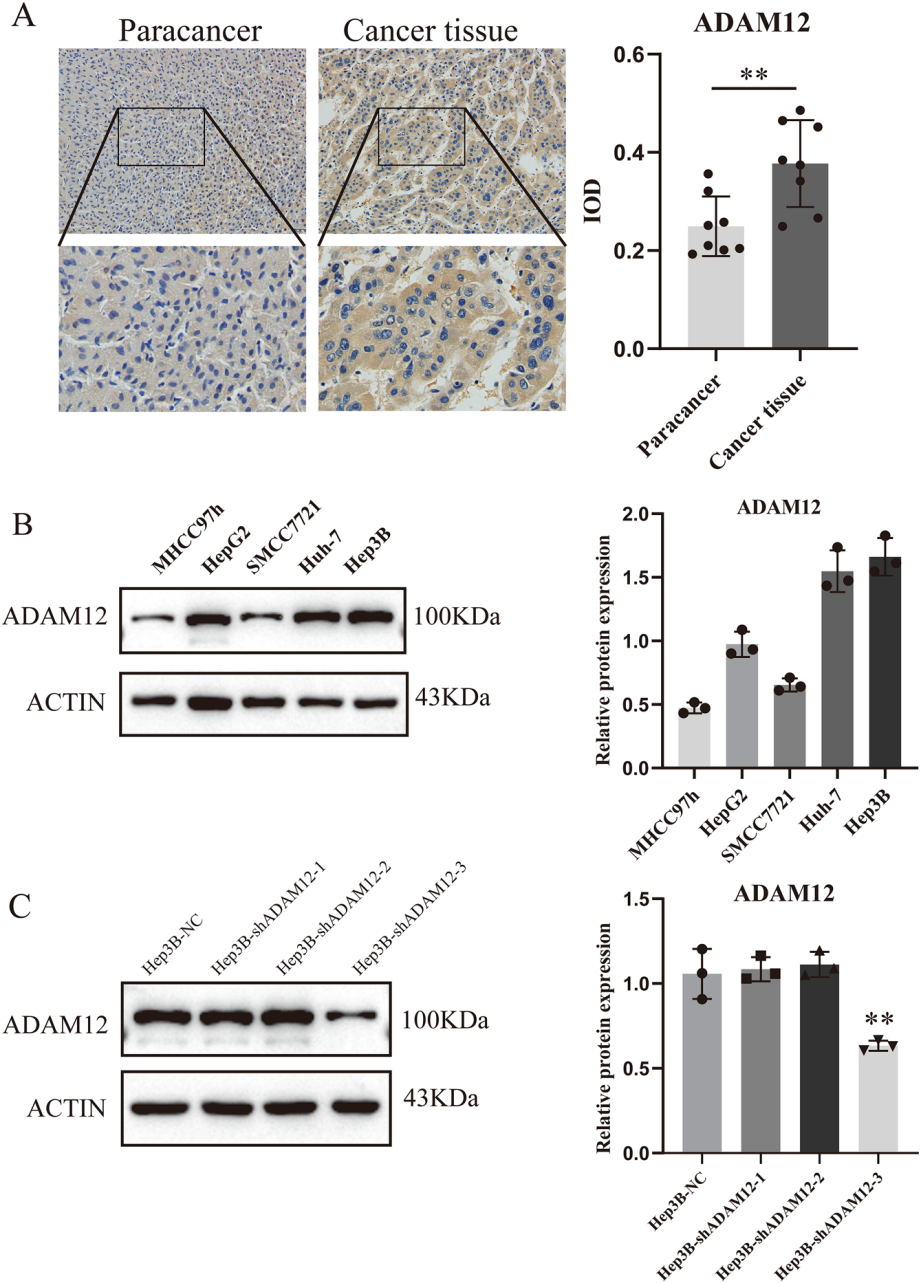
Differential expression of ADAM12 in hepatocellular carcinoma tissues and hepatocellular carcinoma cells. (**A**) The expression of ADAM12 in cancer tissues and adjacent tissues was detected by IHC (n = 8). (**B**) Expression level of the *ADAM12* gene in different hepatocellular carcinoma cells. (**C**) The validity of the ADAM12 gene shRNA plasmid was verified by Western blot analysis. Magnification, 100 × ; scale bar, 20 μm. The data are presented as the mean ± SD of three independent experiments. The display of cropped gels and blots in the picture and original blots/gels are presented in Supplementary PDF. *p < 0.05, **p < 0.01 and ***p < 0.001 indicate significance.

To verify the bioinformatics analysis results and determine the correlation between ADAM12 gene expression and liver cancer, we first compared the expression of ADAM12 in cancer tissues and adjacent tissues taken from liver cancer patients. The results showed that the expression level of the ADAM12 protein in the cancer tissues was significantly higher than that in the adjacent tissues (Fig. 6A, *p* < 0.05). Next, we detected the protein expression level of ADAM12 in different HCC cell lines. A Western blot analysis indicated that the ADAM12 protein expression level was relatively high in Huh-7 cells and Hep3B cells (Fig. 6B, *p* < 0.05). In addition, we validated shRNA interference sequences. The results showed that the third interference sequence tested was effective in inhibiting ADAM12 expression (Fig. 6C, *p* < 0.05).

### Knocking down ADAM12 expression inhibited the proliferation of HCC cells and blocked the G1/S transition

**Figure 7:**
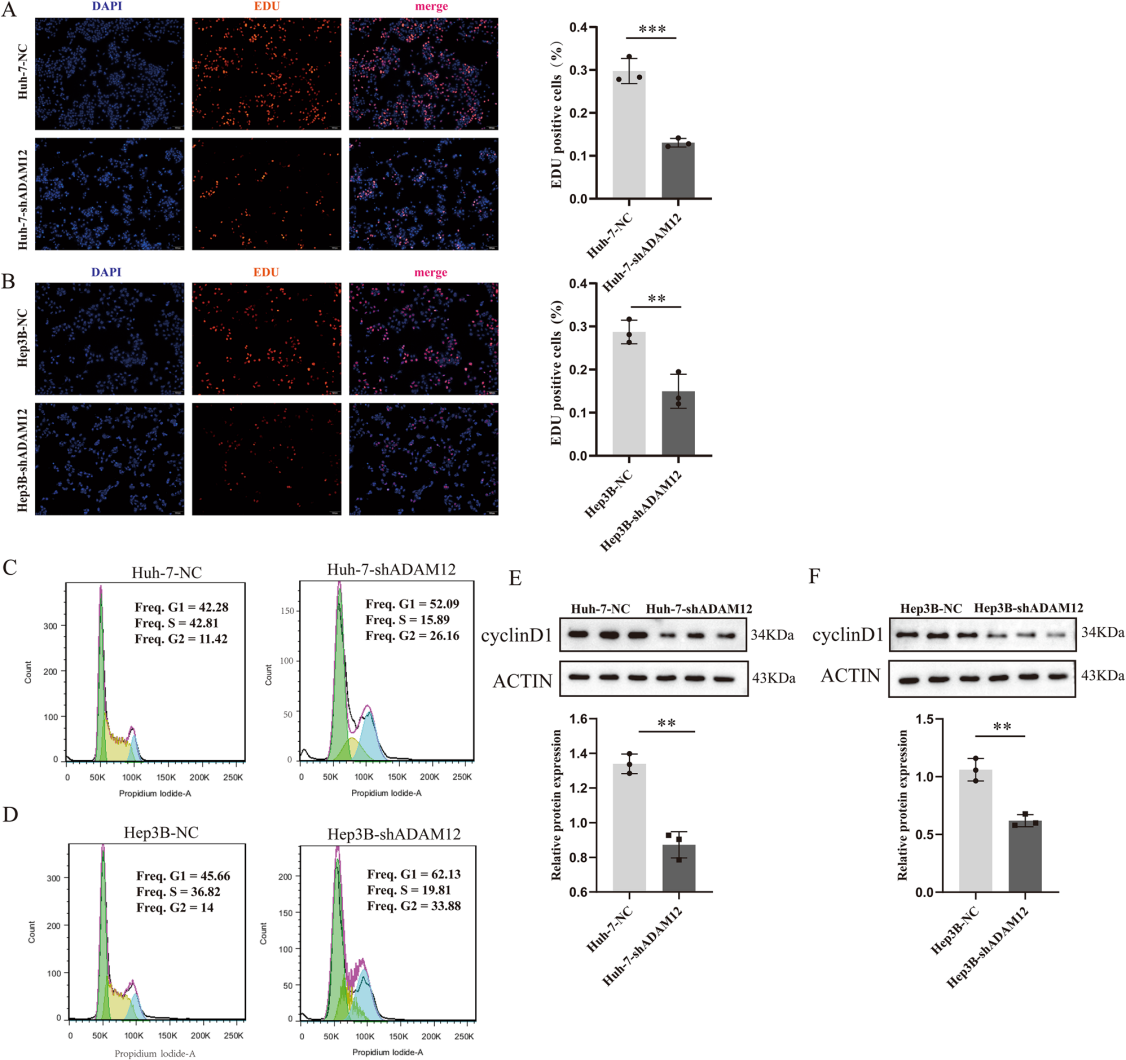
Knockdown of ADAM12 expression inhibited the proliferation of HCC cells and blocked the G1/S transition. (**A, B**) The proliferation rate of hepatocellular carcinoma cells transfected with control or ADAM12 shRNA was detected by EdU proliferation. (**C, D**) Detection of cell cycle after low expression of ADAM12. (**E, F**) The key cell cycle proteins were detected by western blotting. Magnification, 100 × ; scale bar, 100 μm. Each experiment (treatment) was performed in triplicate. The data are shown as the means ± SD. The display of cropped gels and blots in the picture and original blots/gels are presented in Supplementary PDF. *p < 0.05, **p < 0.01 and ***p < 0.001 indicate significance.

To further clarify the role of ADAM12 expression in the progression of HCC, we performed related functional experiments. An EdU proliferation experiment was performed to determine whether the expression level of ADAM12 affected the cell proliferation rate. The results showed that inhibition of ADAM12 expression resulted in a decreased cell proliferation rate compared with that of the control group (Fig. 7A, B). To further explore the specific reasons that the ADAM12 gene affects the proliferation and viability of liver cancer cells, we carried out cell cycle experiments. The results showed that low ADAM12 gene expression blocked the transition of liver cancer cells from the G1 phase to the S phase (Fig. 7C, D). CyclinD1 is a key protein affecting the G1 phase transition^29^. Our results showed that the expression level of cyclin D1 decreased after ADAM12 expression was reduced (Fig. 7E, F). These results suggested that low expression of ADAM12 may inhibit the proliferation of hepatoma cells by suppressing cell cycle progression, further suggesting that the *ADAM12* gene is related to poor prognosis in HCC.

### Knockdown of ADAM12 expression inhibited the expression levels of TGF-β and Notch signalling pathway components

**Figure 8:**
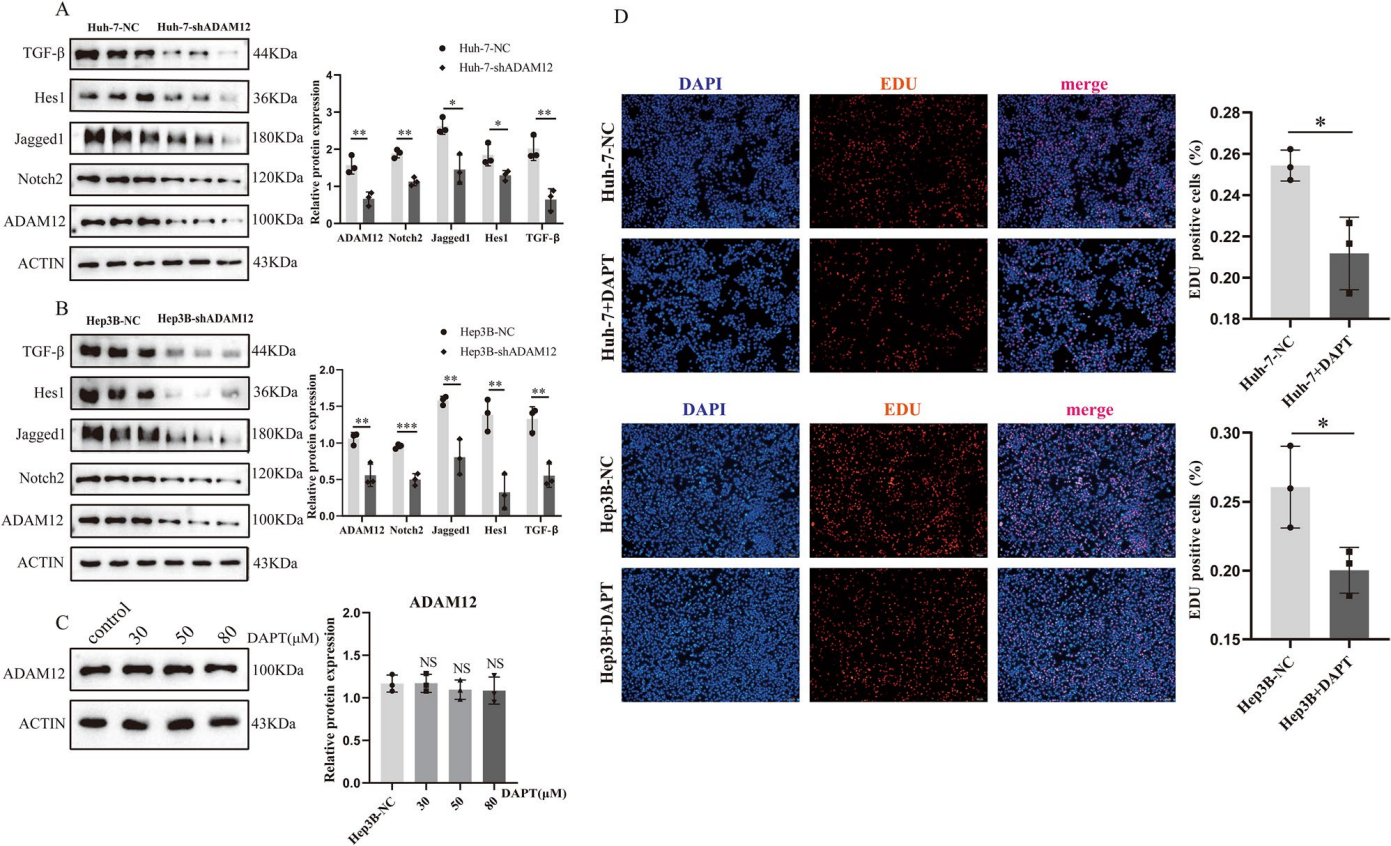
Knockdown of *ADAM12* gene expression inhibited Notch and TGF-β signalling pathway activation in hepatocellular carcinoma cells. (**A, B**) Relative protein expression levels of Notch signalling pathway markers (Notch2, Jagged1, and Hes1) and a TGF-β signalling pathway marker (TGF-β) were determined by Western blotting. (**C**) The expression of ADAM12 was not affected in cells treated with different concentrations of DAPT inhibitors for 48 h (DAPT, 30 μM, 50 μM, or 80 μM). (**D**) Compared with the control group, the DAPT inhibitor group exhibited inhibited liver cancer cell proliferation (DAPT, 50 μM). Magnification, 100 × ; scale bar, 100 μm. The data are presented as the mean ± SD of three independent experiments. The display of cropped gels and blots in the picture and original blots/gels are presented in Supplementary PDF. *p < 0.05, **p < 0.01 and ***p < 0.001 indicate significance.

As shown in Fig. 4, the GSEA revealed that in the *ADAM12* gene high-expression group, the most significantly enriched pathway was the Notch signalling pathway. To confirm this finding, specific ADAM12 shRNA was first transfected into cells to inhibit ADAM12 expression. The results showed that the expression levels of key Notch signalling pathway proteins Notch2, Hes1 and Jagged1 were significantly downregulated in the ADAM12 low expression group compared with the control group, suggesting that the Notch pathway was blocked (Fig. 8A, B). Similarly, the expression level of TGF-β was also decreased upon *ADAM12* gene expression inhibition (Fig. 8A, B). The γ-secretase inhibitor DAPT is a potent inhibitor of the Notch signalling pathway, and therefore, it was added to the cell cultures; however, inhibition of Notch signalling did not change the expression of ADAM12 (Fig. 8C), but it inhibited the proliferation of liver cancer cells (Fig. 8D). Taken together, these data indicated that the ADAM12 gene may be involved in the occurrence and development of HCC through its effects on the Notch signalling pathway. The in vitro experimental results were consistent with those of the bioinformatics analyses.

## Discussion

Primary liver cancer is caused by various complex factors; however, it primarily results from increased liver cell damage. Liver fibrosis is a chronic pathological process caused by excessive deposition of ECM, which induces liver injury and subsequent changes in the microenvironment to induce primary liver cancer occurrence and development^30,31^. Considering the role of liver fibrosis in liver cancer, Elsharkawy's team proposed the "inflammation-fibrosis-cancer axis" theory in 2007^32^. Notably, the relationship between ADAM12 expression and liver cancer has rarely been reported; however, ADAM12 has been shown to promote liver fibrosis. Studies have indicated that ADAM1*2* is negligibly expressed in normal liver tissue, but after it is highly expressed, ADAM12 activates hepatic stellate cells by activating transforming growth factor-β (TGF-β)^25^. Moreover, Nathalie's team proposed that *RACK1* is a chaperone for ADAM12 promotion of liver fibrosis^22^. Therefore, the effect of ADAM12 expression on hepatic fibrosis implies that ADAM12 might be closely related to the occurrence and development of liver cancer.

We analysed the integrated data and discovered that the expression level of the *ADAM12* gene in primary liver cancer tissues was significantly higher than that in paracancerous tissues. The survival analysis and logistic regression analysis revealed that patients with high *ADAM12* gene expression exhibited a low survival rate and poor prognosis. The multivariate Cox regression analysis showed that the *ADAM12* gene is an independent risk factor for HCC. Tumour staging (TNM) is used to evaluate the number and location of malignant tumours in vivo, and it is used to guide clinical treatment, to a certain extent, since each stage (I to IV) is associated with different prognostic characteristics^33,34^. In the present study, the expression level of the *ADAM12* gene was significantly and positively correlated with tumour size and pathological stage, indicating that the *ADAM12* gene can potentially be used as an indicator of liver cancer stage.

Excessive cell proliferation and rapid metastasis are typical features of the sustained development of malignant tumours; these processes are regulated by multiple signalling pathways in vivo^35–37^. We found that highly expressed *ADAM12* was primarily enriched in the Notch, GnRH, Hedgehog, TGF-β, JAK/STAT, MAPK, Calcium, Neurotrophin, and Fc Epsilon signalling pathways. Previous studies have implicated Notch in the proliferation, migration, and metastasis of various cancer cells^38–40^. However, Notch signalling may play a contradictory role due to the interaction between pathway regulatory mechanisms and the microenvironment^41,42^. However, several lines of evidence have indicated that Notch signalling pathways are significantly associated with cirrhosis, liver fibrosis and HCC^43–45^. Similar to the Notch pathway, the TGF-β signalling pathway plays a dual role in the development of HCC and inhibits the early development of HCC^46^. Nonetheless, hepatoma carcinoma cells undergo the EMT after responding to TGF-β, and therefore, the migration and invasion of liver cancer cells are increased^47,48^. Importantly, our study validated the association of *ADAM12* gene expression with the Notch and TGF-β signalling pathways. Viral hepatitis and NASH are important causes of liver cancer development. Liver fibrosis due to viral hepatitis and NASH has been shown to be a risk factor for the development of hepatocellular carcinoma^49–51^. It has been suggested that ADAM12 is significantly associated with liver fibrosis^52^. The results of this study showed that ADAM12 was positively correlated with the expression of TGF-β (a marker of fibrosis) in liver cancer. In addition, activation of the Notch pathway increases the rate of liver fibrosis^53^. Therefore, the expression of the ADAM12 gene in the liver may promote the progression of hepatocellular carcinoma through the formation of liver fibrosis, but the data are not conclusive. In contrast to the complex roles of the Notch and TGF-β signalling pathways, the Hedgehog signalling pathway has been found to be consistently abnormally activated in association with tumour progression, metastasis, and drug resistance^54–56^. In HCC, the high expression of SMO and GLI1, members of the Hedgehog signalling pathway, directly triggers the formation of larger tumours and is significantly associated with recurrence^57,58^. In addition, the JAK/STAT and MAPK signalling pathways are associated with antitumour immunity^59^. Both the JAK/STAT and MAPK pathways have been confirmed to be crucial targets in related studies of HCC resistance^60,61^.

In recent years, tumour immune evasion has been a hot topic in research related to tumour treatment. Several immune cells, including macrophages, T cells, and autonomous NK cells reside in the microenvironment of tumour tissues. These cells directly or indirectly affect the tumour cell microenvironment and regulate tumour cell behaviour. For instance, in hepatocellular carcinoma, the antigen-specificity of invasive T cells is highly correlated with tumour control that is specifically manifested as CD8 + T cells, which are associated with an effective antitumour response^62^. Notably, by upregulating IL-10 expression, HIG2 facilitates HCC evade the killing induced by NK cells, promoting HCC cell recurrence and metastasis^63^. Because of these outcomes, immune cell therapy in hepatocellular carcinoma has broad application prospects. The expression of ADAM12 may be associated with immune cell infiltration in hepatocellular carcinoma; these invading cells include macrophages, immature dendritic cells, follicular T cells, and helper T cells. These findings may provide potential insights into the use of immunotherapy for patients with liver cancer.

## Conclusion

In conclusion, we used bioinformatics to explore the close correlation between high *ADAM12* gene expression and hepatocellular carcinoma. In addition, we evaluated the feasibility of *ADAM12* gene expression as a prognostic factor for hepatocellular carcinoma and predicted the possible ways that ADAM12 affects the progression of liver cancer, thereby providing a reliable basis for the prevention and prognosis of hepatocellular carcinoma. Finally, inhibition of ADAM12 gene expression significantly blocked the G1 phase transition and thus reduced cell proliferation in our experiments. Furthermore, knocking down ADAM12 expression inhibited Notch pathway activation and reduced the TGF-β expression level. Although Huh-7 and Hep3B cell lines were mainly used in the experiments, we will verify the results with human and murine HCC tissues in future studies. In summary, we speculate that ADAM12 exhibits great potential for promoting HCC progression, leading to a poor prognosis for patients with HCC.

## Methods

### Data mining the TCGA database

The gene expression data and basic clinical characteristic data, based on 50 samples of normal liver tissue and 374 samples of liver cancer tissue (workflow type, HTSeq-FPKM), were downloaded from the TCGA website (https://portal.gdc.cancer.gov/repository), which is a publicly available database. The expression information of the *ADAM12* gene was obtained through high-throughput sequencing of TCGA data. First, an analysis of differential *ADAM12* gene expression was performed, and the results are presented with a box diagram and paired difference diagram. Ultimately, the RNA-Seq HTSeq-Counts gene expression data for liver cancer patients and the clinical data were analysed with R software (version 3.6.3).

### Gene set enrichment analysis (GSEA)

GSEA software was downloaded from the GSEA website (GSEA V4.1.0; https://www.gsea-msigdb.org/gsea/index.jsp). Based on the median *ADAM12* gene expression level, the patient data were categorized into high and low groups. Then, these TCGA data were prepared in text format and imported into GSEA software. Notably, the gene set arrangements were repeated 1,000 times for each analysis. For the GSEA, the P value, FDR value, ES value, and NES value were mainly assessed; these are widely recognized screening parameters. Gene enrichment sequencing was first performed based on the NES values; then, in the gene enrichment analysis, the genes with expression differences that met the *p* < 0.05 and FDR < 25% criteria were considered to be significantly differentially expressed^64^.

### Survival analysis

The Kaplan–Meier Plotter (http://kmplot.com/analysis/) was used to analyse the prognosis of 25 types of tumours. The *ADAM12* gene in hepatocellular carcinoma was analysed using this website, and overall survival was estimated by calculating logarithmic grade P values and hazard ratios (HRs).

### Cell culture

Huh-7 and HepG2 human hepatoma cell lines were purchased from the Cell Bank of the Chinese Academy of Sciences of Shanghai. SMCC7721, Hep3B and MHCC97h human hepatoma cell lines were gifts from Prof. Zhilin Qi, Department of Biochemistry and Molecular Biology, School of Wannan Medical College. All the cells were authenticated by STR profiling. The Huh-7, HepG2, Hep3B and MHCC97h cells were cultured in DMEM (Gibco, USA); the SMCC7721 cells were cultured in RPMI-1640 medium (Gibco, USA). All the cells were cultured in the suitable medium containing 10% foetal bovine serum (FBS; Lonsera, South America) at 37 °C with 5% CO_2_.

### Cell transfection^65^

ADAM12 shRNA interference (pRNAT-U6.1/Neo) and negative plasmids were produced by Ruibo Biological Technology Co., Ltd. (Guangzhou, China). Cells were transfected with short hairpin RNA (shRNA) with the following sequence: 5′-GGGTTCACGAGTGTGCAAT-3′ against ADAM12. Briefly, cells were plated into 12-well cell culture plates. When the cells reached 60%-80% confluence, transfection was carried out using Lipofectamine® 3000 Reagent (Thermo Fisher Scientific, Inc.) according to the manufacturer's instructions. The transfection efficiency was detected by Western blotting. ImageJ version 1.52 software (National Institutes of Health) was used for the densitometry analysis.

### EdU assay^65^

The proliferation ability of cells was detected with an EdU assay kit following the protocol provided by the manufacturer. In brief, cells were seeded into 24-well plates. After adherence, the cells were incubated with EdU solution for 2 h. Following three washes with PBS, the cells were fixed with 4% paraformaldehyde, neutralized with glycine, and reacted with Apollo® fluorescence dyes. Finally, Hoechst 33,342 reaction solution was used to stain nuclei. The cells were observed by inverted fluorescence microscopy (Olympus microscope, Tokyo, Japan) at 100 × magnification. The proportion of EdU-positive cells (red fluorescence) to Hoechst-stained cells (blue fluorescence) was calculated. The results were analysed using ImageJ version 1.52 software.

### Western blot analysis

The cells were dissolved in RIPA lysis buffer and PMSF (RIPA:PMSF = 100:1, v/v), and cell lysates were collected and then centrifuged for 10 min at 12,500 g. The protein concentration was measured with an ND2000 microspectrophotometer (Thermo Fisher Scientific Inc., Waltham, MA, USA). Proteins were separated by SDS–PAGE (10% gels), transferred to PVDF membranes, blocked with 5% free fat milk at room temperature for 2 h and incubated with primary antibody overnight in a 4° refrigerator. The next day, the membranes were incubated with the corresponding secondary antibody at room temperature for 2 h. ECL (chemiluminescence) fluid and an imaging system were used for exposure and data analysis. Protein expression levels were semiquantified by ImageJ version 1.52 software. The following primary antibodies were used: anti-ADAM12 (China ABclonal/A7940 1:2000), anti-ACTIN (US Sigma A1978 1:5000), anti-Jagged1 (US CST/2620S 1:1000), anti-Notch2 (China ABclonal/A0560 1:2000), anti-Hes1 (US CST/11988S 1:1000) and anti-TGF-β (China ABclonal/A2124 1:2000).

### Immunohistochemistry (IHC)

Eight pairs of clinical liver cancer samples were collected, and paraffin sections were obtained from the Department of Pathology, Yijishan Hospital, Wannan Medical College. The expression of ADAM12 in the liver cancer tissues and adjacent tissues was detected by IHC. First, xylene was used to deparaffinize and a diluted alcohol gradient was used for hydration. Tissue sections were treated for antigen retrieval with citrate antigen retrieval buffer. Then, the tissue was placed in 3% hydrogen peroxide to block endogenous peroxidase and blocked with serum for 30 min. The tissue was incubated with anti-ADAM12 antibody overnight at 4 °C. The next day, labelling with the corresponding secondary antibody was performed, followed by DAB colour development. Finally, the stained tissue was visualized with a light microscope.

### Cell cycle analysis

Cells were collected according to kit instructions (China KGI, Cat: KGA511) and immobilized overnight with a final concentration of 70% ethanol. Next, the cells were washed three times with PBS and centrifuged to remove the supernatant. Finally, the cells were stained according to the instructions of a cell cycle assay kit and maintained in the dark for 30 min at room temperature. A flow cytometer (BD, USA) was used to detect the cell cycle distribution.

### Statistical analysis

R software (version Rx64 V3.6.3) was used for statistical analysis. Wilcoxon rank-sum test was performed to analyse the difference in the *ADAM12* gene expression between the normal liver tissue and liver cancer tissue. In addition, Wilcoxon signed-rank test, logistic regression, and Kruskal–Wallis tests were performed to analyse the relationship between *ADAM12* gene expression and clinicopathological features, excluding patients with incomplete clinical data. The overall survival curve was plotted on the basis of a Kaplan–Meier analysis, and univariate and multivariate Cox regression analyses were performed to assess the effects of the *ADAM12* gene and clinical characteristics on overall survival. Experiments were performed in triplicate, and the data are shown as the means ± SD. The statistical significance of differences was determined by Student's t test for comparisons of two groups. *p* < 0.05 was considered to be statistically significant.

### Ethics approval

This study was approved by the Ethics Committee of Scientific Research and New Technology of Yijishan Hospital of Wannan Medical College with the approval number of 202095, and obtained informed consent from all patients who participated in this study. All methods were performed in accordance with the Helsinki declaration guidelines and regulations.

### Consent for publication

We agree to publish the manuscript.

## Supplementary Information

Supplementary Information 1.

Supplementary Information 2.

## Data Availability

All data generated or analysed for this study are included in this article. Further details are available from the corresponding author upon request. Patient data that supported the findings of this study are available in The Cancer Genome Atlas (TCGA) datasets at https://portal.gdc.cancer.gov/repository.

## Abbreviations

ADAM12: Disintegrin and metalloproteinase 12
TCGA: The Cancer Genome Atlas database
HCC: Hepatocellular carcinoma
FBS: Foetal bovine serum
GSEA: Gene set enrichment analysis
HR: Hazard ratio
CHOL: Cholangiocarcinoma
TGF-β: Transforming growth factor-β
TNM: Tumour staging
KEGG: Kyoto Encyclopedia of Genes and Genomes
