# Supplementary material for: ADAM12 is an independent predictor of poor prognosis in liver cancer
Source: Sci Rep. 2022 Apr 22;12:6634. doi: 10.1038/s41598-022-10608-y (PMC9033838; doi:10.1038/s41598-022-10608-y)
Supplement: Supplementary file 1 — Supplementary Information 1. [file 41598_2022_10608_MOESM1_ESM.pdf]

Fig6

Original blots in supplementary information

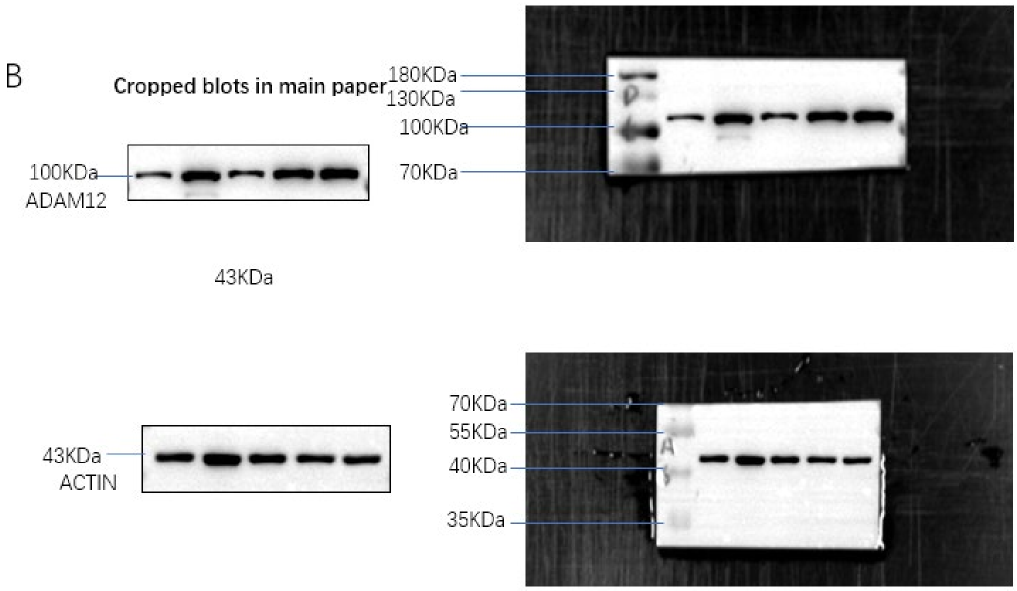

Fig6

Original blots in supplementary information

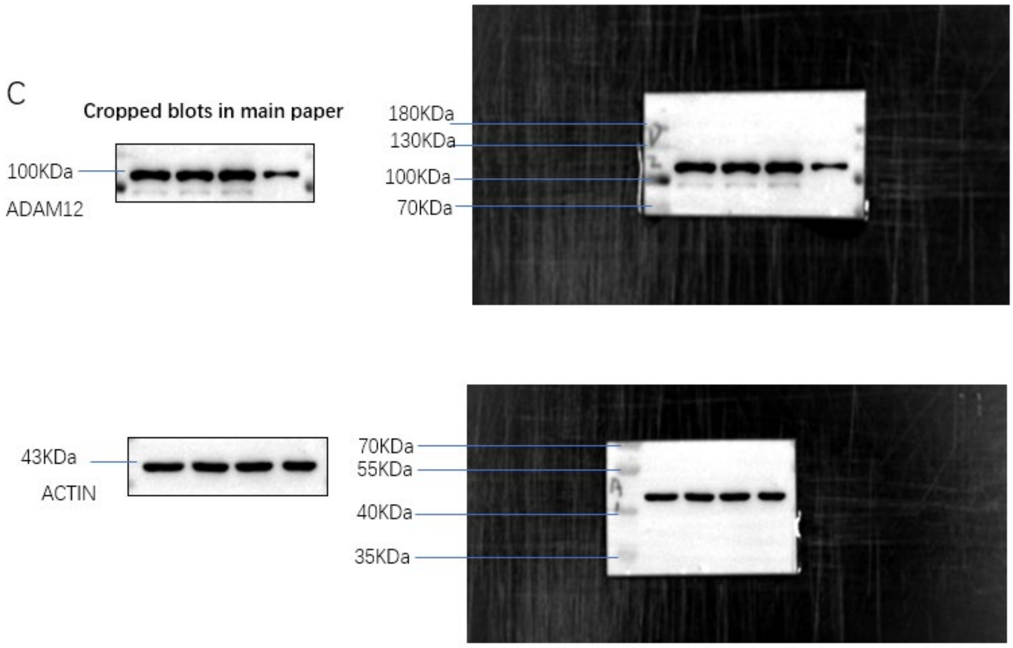

Fig7

Original blots in supplementary information

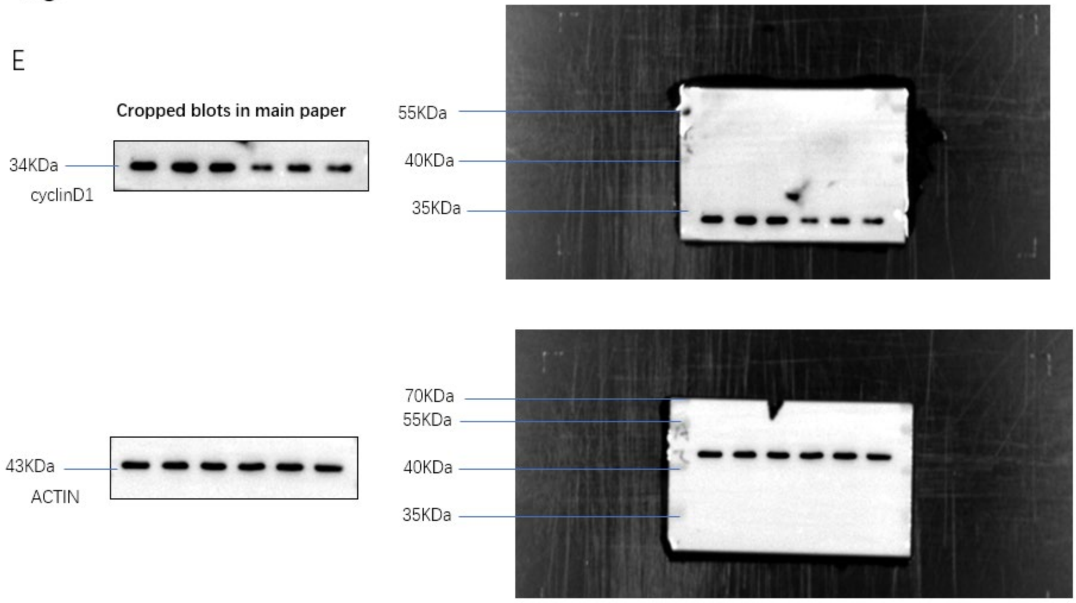

Fig7

Original blots in supplementary information

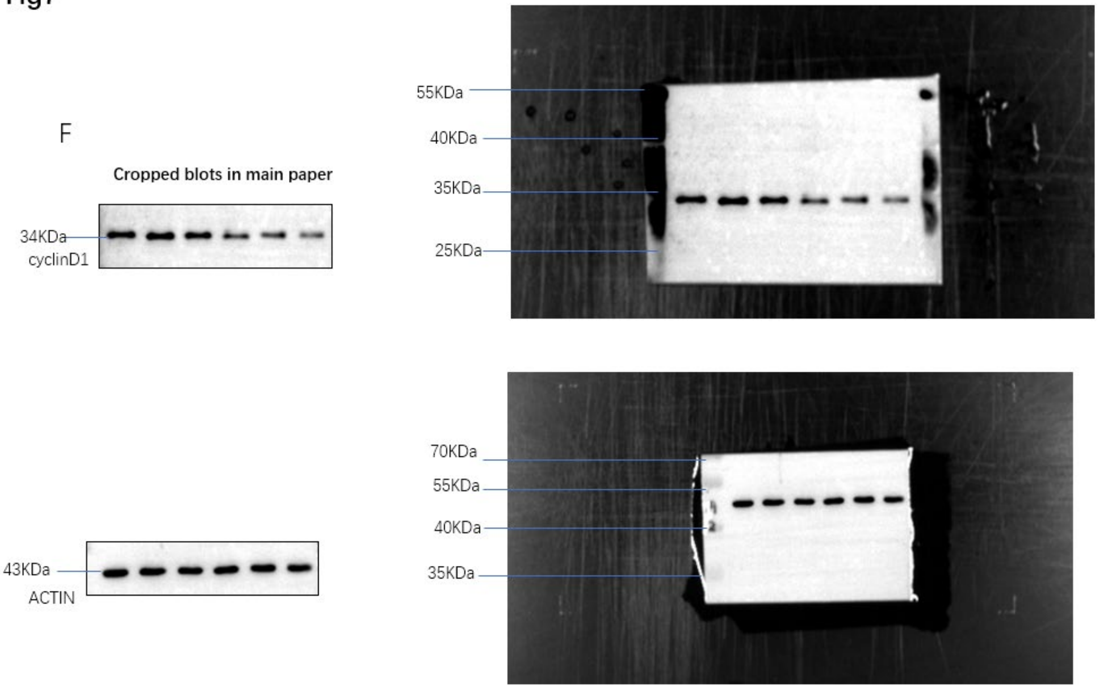

Fig8

Original blots in supplementary information

A

Cropped blots in main paper

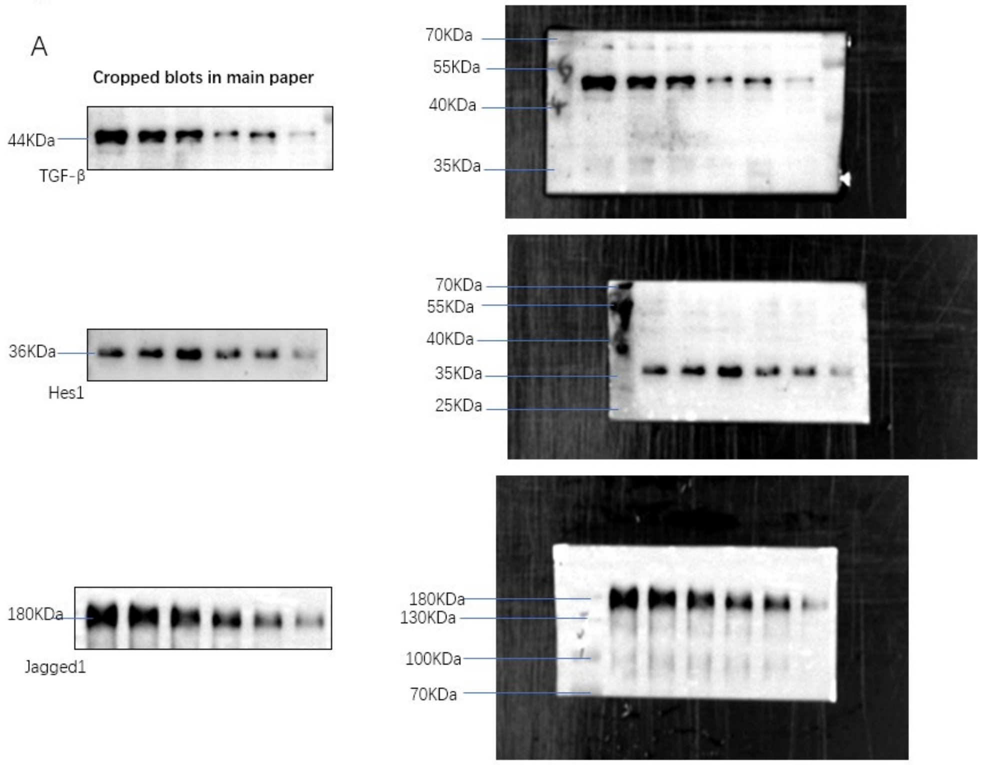

Fig8

Original blots in supplementary information

A

Cropped blots in main paper

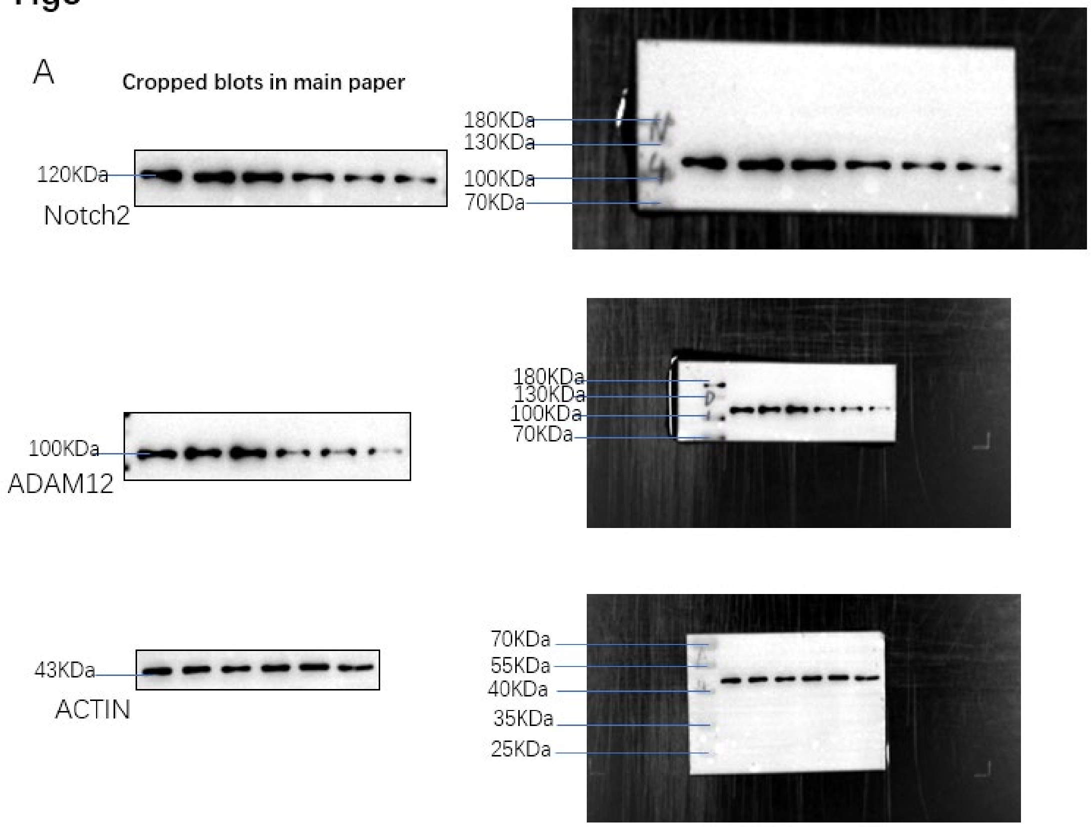

Fig8

Original blots in supplementary information

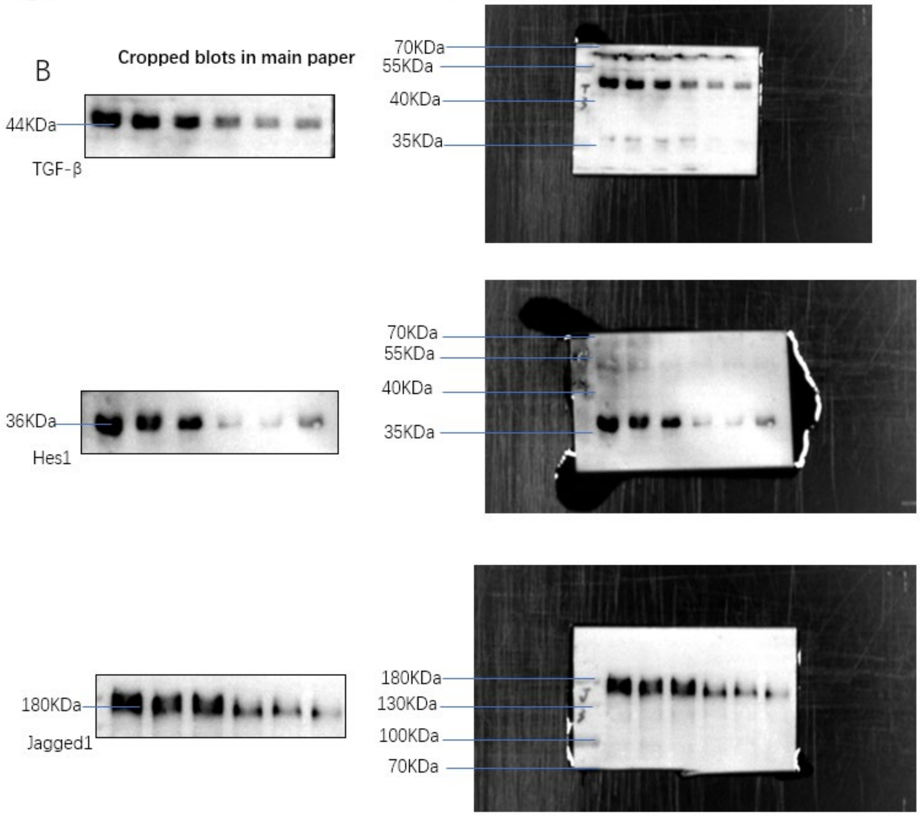

Fig8

Original blots in supplementary information

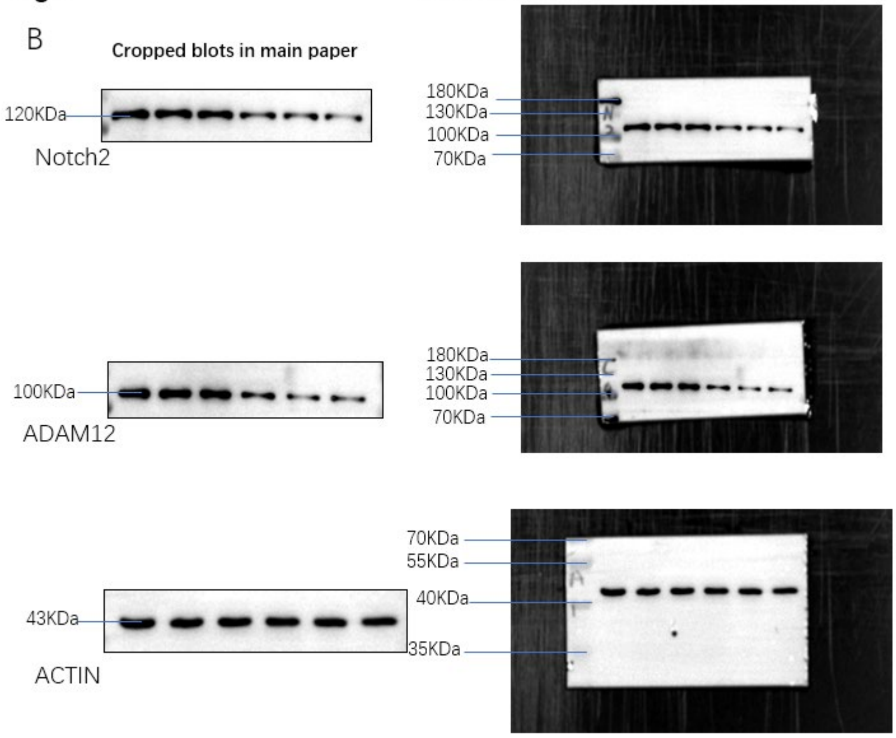

Fig8

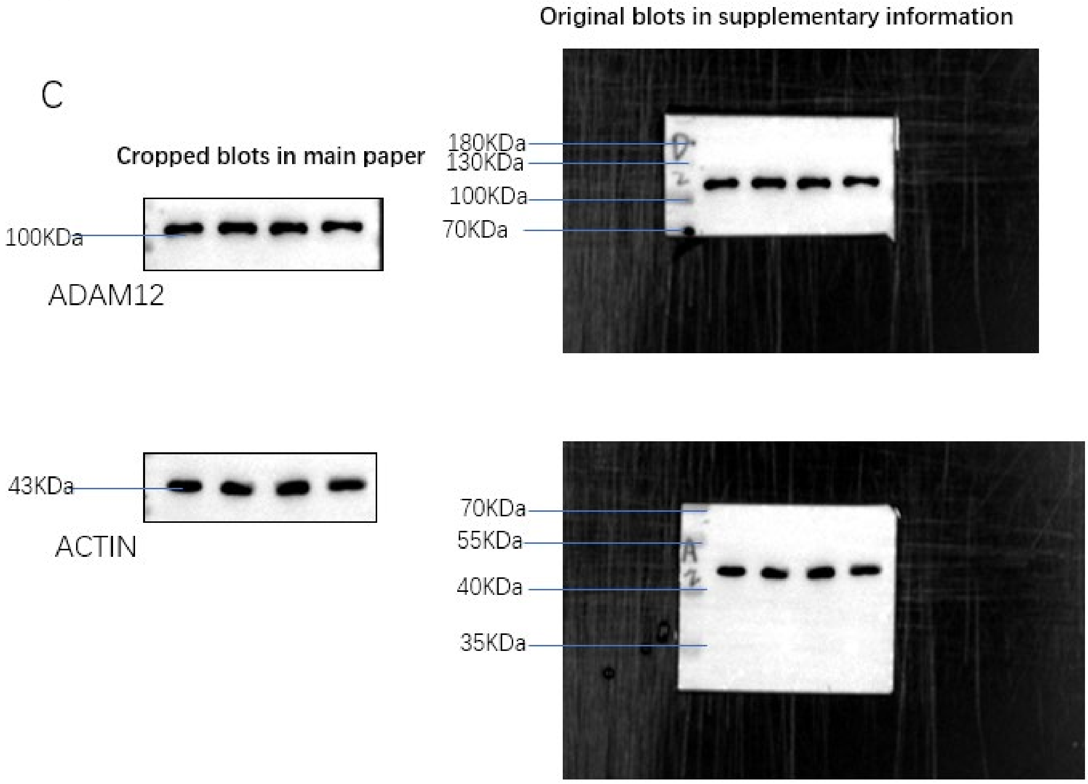

Original blots in supplementary information
C
Cropped blots in main paper
100KDa
ADAM12
180KDa
130KDa
100KDa
70KDa
43KDa
ACTIN
70KDa
55KDa
40KDa
35KDa
